# Disease causality extraction based on lexical semantics and document-clause frequency from biomedical literature

**DOI:** 10.1186/s12911-017-0448-y

**Published:** 2017-05-18

**Authors:** Dong-gi Lee, Hyunjung Shin

**Affiliations:** Department of Industrial Engineering, Ajou University, 206 Worldcup-ro, Yeongtong-gu, Suwon 16499 South Korea

**Keywords:** Disease causality, Text mining, Lexical semantics, Document-clause frequency

## Abstract

**Background:**

Recently, research on human disease network has succeeded and has become an aid in figuring out the relationship between various diseases. In most disease networks, however, the relationship between diseases has been simply represented as an association. This representation results in the difficulty of identifying prior diseases and their influence on posterior diseases. In this paper, we propose a causal disease network that implements disease causality through text mining on biomedical literature.

**Methods:**

To identify the causality between diseases, the proposed method includes two schemes: the first is the lexicon-based causality term strength, which provides the causal strength on a variety of causality terms based on lexicon analysis. The second is the frequency-based causality strength, which determines the direction and strength of causality based on document and clause frequencies in the literature.

**Results:**

We applied the proposed method to 6,617,833 PubMed literature, and chose 195 diseases to construct a causal disease network. From all possible pairs of disease nodes in the network, 1011 causal pairs of 149 diseases were extracted. The resulting network was compared with that of a previous study. In terms of both coverage and quality, the proposed method showed outperforming results; it determined 2.7 times more causalities and showed higher correlation with associated diseases than the existing method.

**Conclusions:**

This research has novelty in which the proposed method circumvents the limitations of time and cost in applying all possible causalities in biological experiments and it is a more advanced text mining technique by defining the concepts of causality term strength.

**Electronic supplementary material:**

The online version of this article (doi:10.1186/s12911-017-0448-y) contains supplementary material, which is available to authorized users.

## Background

Research on human diseases has been a major issue in biology and medical fields. Research activities on these subjects were carried out based on genetic, biological, and epidemiological information [1–3] in the past, and success in multi-omics approaches has shed light on recent researches on human disease network. For instance, the work of Goh et al. [4], which was regarded as an initiative work on human disease network, constructed a disease network based on genes that are shared by two diseases. On the other hand, Zhang et al. [5], defined disease association using protein interaction. Lee et al. [6] used metabolic pathway to relate two diseases by checking if a disease-related gene exists in the same pathway. Further, disease association in the network has been further extended to clinical or medical information. Folino et al. [7] and Hidalgo et al. [8] discovered disease association from coexisting diseases in clinical records. On the other hand, Zhou et al. [9] proposed a method that determines disease association from shared symptoms. More research on disease association can be found in [10] and [11].

In the previous approaches, however, most disease networks have no direction. The limitation was mainly due to lack of information that determines causal relationship between diseases. Causal relationship of diseases means that when one disease has occurred, other related diseases could co-occur.

Disease causality can be used in many ways such as preventing prior disease in advance or treating posterior diseases once the prior one occurs. For example, when hepatitis has occurred to a patient, liver cirrhosis and hepatocellular carcinoma could occur, and the posterior diseases can lead to death of the patient [12, 13]. If we can determine the causal relationship of these diseases, we can apply priority prevention of posterior disease and choose an appropriate treatment method. Therefore, determining not only the association between diseases but also their causalities is very important. For finding disease causalities, Bang et al. [14] proposed a causality modeling by using various biomedical data including gene/protein, clinical, metabolic pathway information to construct a disease causality network.

The sources of disease causality can be obtained by experiments or from clinical reports. In the experimental approach, causality may be determined based on the shared genes from prior–posterior diseases or influencing genes along the metabolic pathways. However, because many genes, pathways, and diseases can be present, we encounter limitations in terms of time and cost in applying all possible causalities in experiments. On the other hand, referencing clinical reports to identify prior–posterior relationship between diseases entails violation of privacy. Most medical records are not open to the public. Therefore, one of the methods of circumventing the difficulties presented above is using biomedical literature officially open to the public. Biomedical literature contains reports on experimental results and clinical comorbidity information on disease causality. Recent advances in text mining can save time and effort when looking through a large amount of documents and help us extract useful information that can be utilized to define causal relationships between diseases. Earlier works using text mining have been performed. Ananiadou et al. [15] used text mining to extract gene or protein relationship information but only to extract the associations of genes or proteins in the molecular biology level. Similar works can also be found in [16–19]. In the clinical level, many researches have been made using text mining that attempt to find the shared phenotypes and symptoms of diseases from documents. The readers are referred to [9, 20, 21]. Furthermore, Xu et al. [22] searched disease-disease risk relationships in the biomedical literature using text mining approach.

**Fig. 1:**
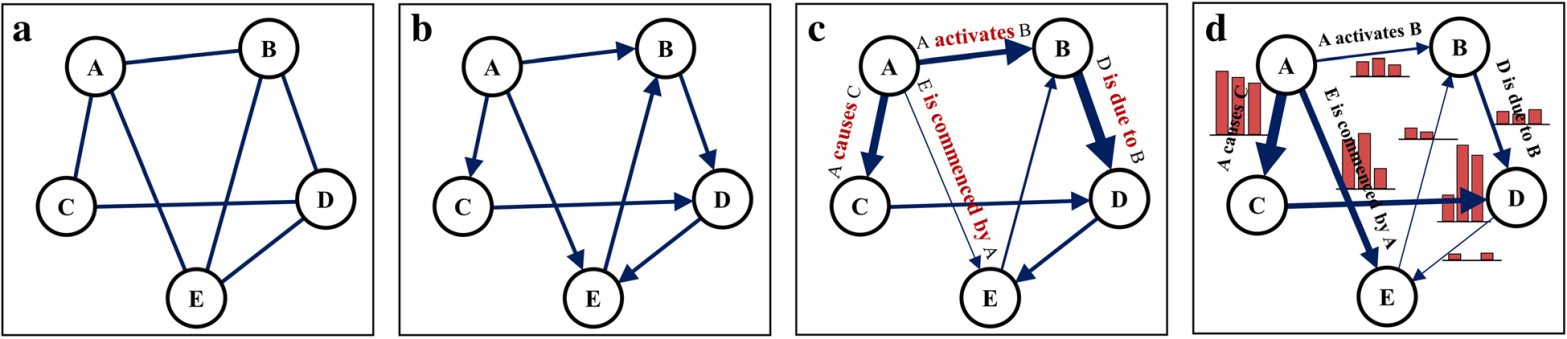
Evolution of disease networks from association to causality: **a** Disease network with association only between the diseases. **b** Simple causal disease network that shows the prior–posterior relationship of diseases. **c** Causal disease network that considers the semantic strength of causality terms based on lexicon analysis. **d** Causal disease network that reflects the different strengths of causality terms and causal frequencies extracted from documents

In this paper, we propose a causal disease network, which constructs disease causality through text mining on biomedical literature. To provide causality between diseases, the proposed method includes two schemes: the first is the lexicon-based causality term strength, which provides causal strength on variety of causality terms based on lexicon analysis. The second is the frequency-based causality strength, which determines the direction and strength of the causality based on document and clause frequencies in the literature. Figure 1 shows a schematic description of evolution of a disease network that contains information from association to causality. In particular, causal disease networks can be laid on a variety of information spectra depending on how finely the directional information is reflected. Figure 1b shows that a simple causal disease network displays only the prior–posterior relationship of diseases. However, Fig. 1c shows that the edges of the network represent the semantic strength of the causality terms based on lexicon analysis. Figure 1d shows that the network reflects both the different strengths of the causality terms and causal frequencies extracted from documents. In the proposed method, we first implement the lexicon-based causality term strength to construct a causal network, as shown in Fig. 1c. Then, we incorporate it into the frequency-based causal strength. The resulting network will then have the most advanced form in the spectrum of causality network, as shown in Fig. 1d.

## Methods

In the proposed method, we define the concepts of causality term strength based on lexical semantics and define the causality frequency based on biomedical literature to discover the causal relationships between diseases, along with their strength and directions.

### Lexicon-based causality term strength

A hint on the causality between diseases can be found in the clause of a sentence in text data. For example, the clause “A causes B” in a sentence denotes causal relationships between A and B. We define these clauses as causality clauses. By searching for causality clauses that describe the relationship of two diseases in biomedical literature, we can extract prior and posterior diseases. More specifically, causality term strength refers to the strength of causal connotation that a causality term exhibits in a causality clause. For example, the term “causes” has larger causal connotation than “tend to cause” in describing a relationship between two diseases. This definition implies that the former term has more reliability than the latter in deducing causal relationships between the diseases.

**Fig. 2:**
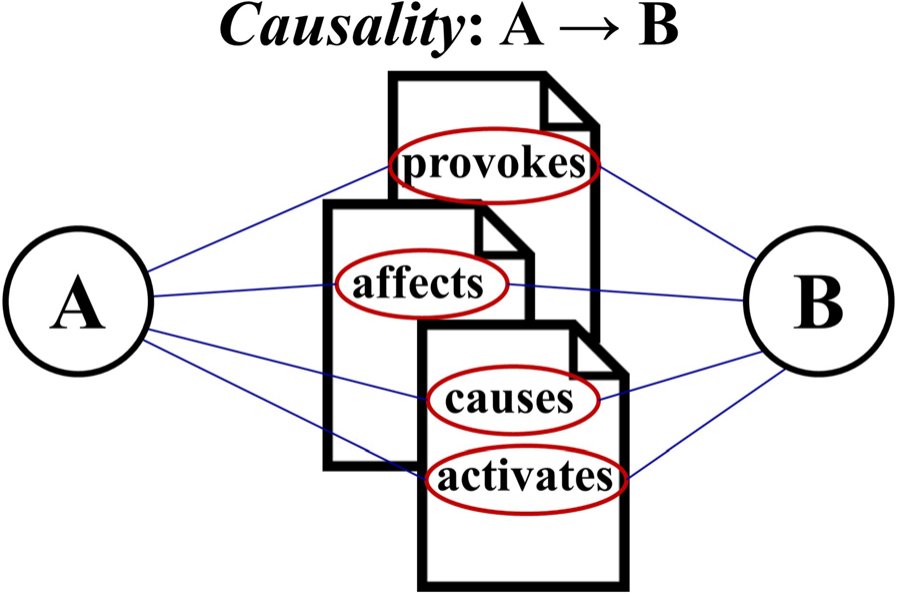
Causal relationship of A and B expressed in various terms

Various types of causality terms exist, and their meanings or degrees of strength differ. Figure 2 shows that the directions of causal relationship of A and B are the same, but the meaning of each term has a different degree of causal strength.

Causality terms can derive their strength according to semantic interpretation. Thus, the stronger the meaning of causal relationship is, the larger is its weight value; when only a simple relationship is indicated, the term has a small weight value. Given a total of $T$ causality terms, ${\upalpha}_{\mathrm{t}}$ gives a weight value for each term, where $t\in T$. We denote ${\upalpha}_{\mathrm{t}}$ as the lexicon-based causality term strength. The weight values are determined through 32 surveys from Anglophones and an advisory of a lexical-semantic and lexicography expert. According to a four-point Likert scale representing “simple relation,” “weak,” “strong,” and “very strong,” ${\upalpha}_{\mathrm{t}}$ has one of the values in $\mathrm{L}=\left\{1,2,3,4\right\}$ and is rescaled to be $0\le {\upalpha}_{\mathrm{t}}\le 1$ as follows:

1 $$ {\upalpha}_{\mathrm{t}}=\frac{{\mathrm{argmax}}_l fre{q}_t(l)}{\left| L\right|} $$

where

$$ f r e{q}_t(l)={\sum}_{i=1}^Q I\left( i, l\right). $$

In (1), $I\left( a, b\right)$ returns “1” if $a= b$; otherwise, it is “0.” $Q$ is the number of surveys. This process applies the major consensus on the Likert scale of those participants who took part in the survey. Table 1 lists the lexicon-based causality term strength (${\upalpha}_t$) assigned to each of the 105 causality terms. The lexicon-based causality term strengths are 33 (32 Anglophones and 1 linguist).

**Table 1:** Causality terms and lexicon-based term strength

Causality Term	${\upalpha}_{\mathrm{t}}$	Causality Term	${\upalpha}_{\mathrm{t}}$	Causality Term	${\upalpha}_{\mathrm{t}}$
activate	1.00	effectuate	1.00	launch	0.75
activated by	0.75	effectuated by	1.00	launched by	0.75
actuate	1.00	elevate	0.75	lead	0.75
actuated by	1.00	elevated by	0.75	led by	0.75
affect	1.00	elicit	0.75	link	0.25
affected by	0.75	elicited by	0.75	made by	1.00
arisen from	0.75	enhance	0.75	make	1.00
arising from	0.75	enhanced by	0.75	originate	0.75
arouse	0.75	entail	0.50	originated by	0.75
associate with	0.25	entailed by	0.75	owe	0.75
attributable to	0.75	fire up	1.00	produce	1.00
attributed to	0.75	fired by	1.00	produced by	1.00
because of	1.00	generate	1.00	promote	0.75
began by	0.75	generated by	1.00	promoted by	0.75
begin	0.75	give birth to	1.00	provoke	0.75
bring	0.75	give rise to	1.00	provoked by	0.75
brought by	0.75	hasten	0.50	relate	0.25
call	0.75	hastened by	0.50	result	1.00
called out by	0.75	implied by	0.50	resulting from	1.00
cause	1.00	imply	0.50	rise	1.00
caused by	1.00	incite	0.75	secondary to	0.50
commence	0.50	incited by	0.75	set off	0.75
commenced by	0.50	induce	0.75	spark	1.00
complicate	0.50	induced by	0.75	sparked by	1.00
complicated by	0.50	infect	0.75	start	1.00
complication	0.50	infected by	1.00	started by	1.00
contribute	0.75	influence	0.75	stem from	1.00
contributed by	0.50	influenced by	0.75	stimulate	0.75
create	1.00	initiate	1.00	stimulated by	0.75
created by	1.00	initiated by	1.00	stir	0.75
develop	1.00	interact	0.25	stirred by	0.75
developed by	1.00	kick up	0.75	trigger	1.00
due to	1.00	kicked up by	0.75	triggered by	1.00
educe	0.75	kindle	0.75	unleash	1.00
educed by	0.75	kindled by	0.75	unleashed by	1.00

### Frequency-based causality strength

The number of documents that phrases causality between diseases is important in determining the reliability of causal relationships. If multiple documents exist that describe the causality between diseases A and B while only a single document exists that describes C and B, the causal relationship of the former is more reliable than that of the latter. Conventional document-frequency-based methods refer to such aspects [23, 24]. The frequency-based causality strength, however, should incorporate the additional aspect of “how many times the causality terms are also clause-wise present.” Thus, the causality frequency discussed here includes calculating the weights of the causality terms that not only considers the document frequency but also incorporates repetition of the causality clauses. Therefore, we propose the document-clause frequency (DCF). DCF counts the occurrence frequency in both the number of documents and the number of clauses. However, even if the number of clauses shows the same occurrence, cases exist when clauses come up from one single document as well as from multiple documents. With regard to which case is more reliable, the answer would be the latter because occurrences in many documents prove causality, which provides more plausibility or reliability than that from a single document occurring multiple times. Equation (2) implements the idea. ${df}_t^{AB}$ indicates the number of documents that expresses the causal relationship of A and B using causality term $t$, and ${cf}_t^{AB}$ indicates the number of clauses.

2 $$ {DCF}_t^{AB}={df}_t^{AB}\cdot \log \left({cf}_t^{AB}+1\right) $$

**Fig. 3:**
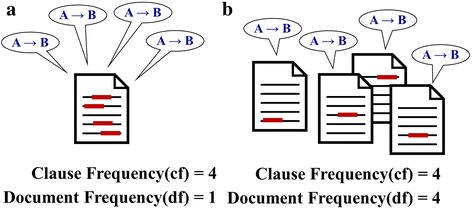
**a** and **b** show the cases where the clauses are the same in terms of clause frequency but are different in terms of document frequency. DCF puts a large value to (**b**): The DCF value of (**a**) is 0.7 and that of (**b**) is 2.8

Figure 3a and b show cases where the clauses are the same in terms of clause frequency but are different in terms of document frequency. Compared with (a), (b) is more plausible and reliable. Therefore, we assign more confidence to case (b) in (2); the DCF values of (a) and (b) are 0.7 and 2.8, respectively.

### Causality weight and direction

Given two diseases, the causality weight and direction are determined by combining the two causality strengths introduced in the previous sections: the lexicon-based causality term strength ${\upalpha}_t$ and the frequency-based causality strength DCF. Equation (3) shows the weight of the causality between diseases A and B.

3 $$ {w}_{AB}={\sum}_{t\in T}\left({\alpha}_t^{AB}\cdot DC{F}_t^{AB}\right) $$

${w}_{AB}$ shows the weight value of (A→B) when A causes B. In the same manner, ${w}_{BA}$ can be calculated if the reverse condition coexists. Using ${w}_{AB}$ and ${w}_{BA}$, the final causal relationship is expressed as

4 $$ \alpha DCFC\left( A, B\right)={w}_{AB}-{w}_{BA}. $$

When $\alpha DCFC>0$, this condition implies that A is more likely to cause B than the reverse case. The amount of influence is quantified by the difference between ${w}_{AB}$ and ${w}_{BA}$. A larger absolute value of $\alpha DCFC$ indicates more confidence on the causality.

## Results and discussion

### Data

To validate the proposed method, we used the data listed in Table 2, which include the sources of diseases, causality terms, and literature. The list of diseases is collected from Medical Subject Headings (MeSH) [25]. MeSH is a thesaurus database for medical areas specified by the United States National Library of Medicine. From the disease category, we collected 4663 diseases. The number of possible pairwise combination among the 4663 diseases reaches 10,869,543, demanding a large amount of time and calculations. To avoid the demanding computational load, we utilized prevalence database HuDiNe [26]. It is a database that shares 13,039,018 patient clinical records that include the number of people who have diseases and comorbid diseases [8]. Referring to HuDiNe, the most prevalent 195 diseases were selected (See Additional file 1: Table S1). To calculate the lexicon-based causality term strength, terms were obtained from WordNet, which is an English vocabulary database that includes synonyms and antonyms [27]. As described earlier, 105 causality terms were extracted. To calculate the frequency-based causality strength, 6,617,833 abstracts were collected from PubMed, which is a biomedical literature database. From the collected abstracts, we extracted 43,248 documents that contained 195 disease names. Then, the documents were parsed into 504,123 sentences using the Stanford Parser [28].

**Table 2:** Data for diseases, causality terms, biomedical literature

	Data Sources	Number of Data
Diseases	MeSH The Medical Subject Headings www.nlm.nih.gov/mesh/	195 out of 4663 diseases
	HuDiNe A site to explore the Human Disease Network www.hudine.neu.edu/	
Causality Terms	WordNet A lexical database for English www.wordnet.princeton.edu/	105 terms
Literature	PubMed Literature US National Library of Medicine National Institutes of Health www.ncbi.nlm.nih.gov/pubmed	6,617,833 abstracts

### Results of causal disease network construction

To demonstrate how the lexicon-based causality term strength and the frequency-based causality strength are applied to 195 selected diseases, we consider an exemplary case of “Hepatitis C and Hepatocellular Carcinoma” and explain the process using them. Hepatitis C is well known to be the cause of liver cirrhosis and hepatocellular carcinoma [12, 13]. In the collected literature data, the relationship between two diseases is expressed using 16 causality terms. Among them, when causality term “*cause*” is used, the clause “Hepatitis C virus causes hepatocellular carcinoma” is extracted from the long sentence “Hepatitis C virus is a hepatotropic RNA virus that causes acute and chronic hepatitis, liver cirrhosis, and hepatocellular carcinoma.”

Table 3 lists other cases that use different terms with their document and clause frequencies. In this table, the second column lists the lexicon-based causality term strength (${\upalpha}_{\mathrm{t}}$), and the third to fifth columns list the document frequency, clause frequency, and DCF value, respectively. Despite the occurrence of the same clause frequency for the two causality terms “induce” and “infect,” their DCF values are different due to the difference in the document frequency. Most of the literature shows Hepatitis C as a prior disease and Hepatocellular Carcinoma as a posterior disease. Using (3), we obtain weight ${\mathrm{w}}_{\mathrm{AB}}=76.84$ (where “A” is Hepatitis C and “B” is Hepatocellular Carcinoma). However, some reports exist that show the opposite case, resulting in ${\mathrm{w}}_{\mathrm{BA}}=8.63$. Nevertheless, the final consequence is obtained by (4) with a value of 68.21 in terms of $\alpha DCFC$. This result implies that the causality direction between the two diseases is determined as “Hepatitis C as a prior disease and Hepatocellular Carcinoma as a posterior disease” with a causal strength of 68.21.

**Table 3:** Causality terms and resulting values for causality extraction between Hepatitis C and Hepatocellular Carcinoma

Causality Term	$\upalpha$	DF	CF	DCF	$\upalpha \bullet \mathrm{D}\mathrm{C}\mathrm{F}$
affect	1.00	1	1	0.30	0.30
cause	1.00	26	27	37.63	37.63
contribute	0.75	1	1	0.30	0.23
develop	1.00	10	10	10.41	10.41
due to	1.00	8	8	7.63	7.63
educe	0.75	1	1	0.30	0.23
effect	1.00	3	3	1.81	1.81
induce	0.75	1	4	0.70	0.52
infect	0.75	4	4	2.80	2.10
lead to	0.75	10	10	10.41	7.81
link	0.25	1	1	0.30	0.08
relate	0.25	21	21	28.19	7.05
result	1.00	1	1	0.30	0.30
rise	1.00	1	1	0.30	0.30
secondary to	0.50	1	1	0.30	0.15
triggered by	1.00	1	1	0.30	0.30
$$ \upalpha \mathrm{DCFC}={\mathrm{w}}_{\mathrm{AB}}-{\mathrm{w}}_{\mathrm{BA}}=76.84-8.63=68.21 $$					

Using a similar process, we extracted 6275 documents and 6838 clauses from 43,248 documents and discovered a causal relationship of 1011 pairs of 149 diseases (the full list of the disease causality pairs are presented in Additional file 1: Table S2). The results of identified disease causalities and codes are accessible in http://www.alphaminers.net. Table 4 lists the top-10-ranked $\alpha DCFC$ pairs. The disease pairs with high $\alpha DCFC$ values are common cases of posterior diseases following the occurrence of prior diseases.

**Table 4:** Top-10-ranked causal disease pairs

Prior Disease	Posterior Disease	$\upalpha \mathrm{DCFC}$
Aneurysm	Hemorrhage	140.38
Glaucoma	Blindness	125.23
Hepatitis C	Liver Diseases	97.58
Thrombosis	Infarction	73.78
Hepatitis C	Carcinoma, Hepatocellular	68.21
Cataract	Blindness	62.04
Pneumonia	Meningitis	59.73
Aneurysm	Subarachnoid Hemorrhage	58.17
Hepatitis B	Carcinoma, Hepatocellular	54.74
Thrombosis	Myocardial Infarction	45.48

**Fig. 4:**
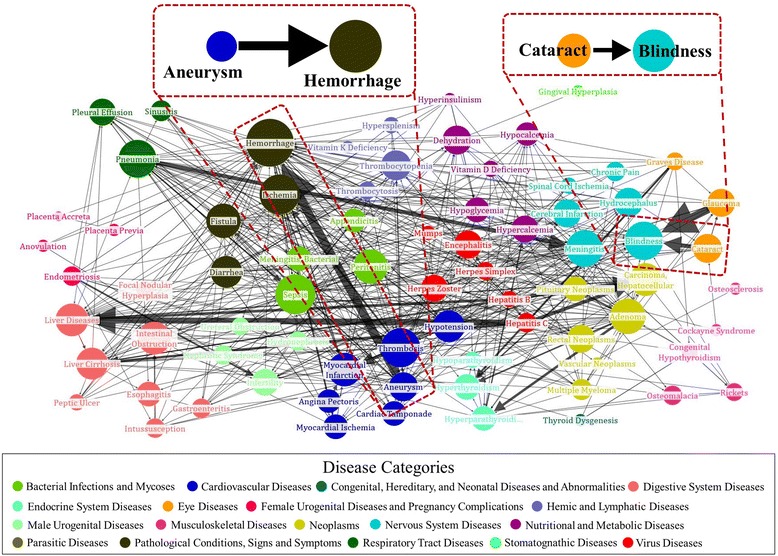
Causal disease network: In the causal disease network, each node denotes a disease, and the color of the node represents the MeSH category. The size of the node is determined by the number of influencing diseases on the disease. The *edge with an arrow* shows the prior–posterior relationship of two diseases, and its width represents the causality strength

Figure 4 shows a subset of the causal disease network of 149 diseases. The subset was obtained, for easier visualization, by selecting upper half of diseases for each category in terms of number of edges. In the network, the nodes indicate diseases, and the direction and width of the edge indicate the $\alpha DCFC$ value. The full network of 149 diseases is presented in Additional file 1: Figure S1. From the magnified portion of the network, we see two pairs of prior and posterior diseases which are (aneurysm → chemorrhage) and (cataract → blindness). From sentences “However, in the case of Moyamoya disease associated with an aneurysm, rupture of the aneurysm should be considered to be a probable cause of subarachnoid hemorrhage” (PMID: 7242821) and “A case of large brainstem hemorrhage resulting from a basilar artery aneurysm is reported” (PMID: 6474340), we could extract that aneurysm causes hemorrhage. Likewise, sentences “Cataract, as the main cause of blindness, will require surgical relief, either in the teaching hospital or preferably in the patient’s locality” (PMID: 14566633) and “The prevalence of blindness (visual acuity [VA] <3/60 in better eye) in 835 people aged ≥ 40 was 1.3% (95% CI 0.5-2.1), of which 36.4% was due to cataract” (PMID: 21780876) allowed us to conclude that the cause of blindness as cataract.

For more enriched analysis, we compared the constructed causal disease network with results in [14]. In [14], they define the causality by exploring the flow of genes of associated metabolic pathways for pairs of diseases. From many of co-identified pairs of causality, one interesting but simple pair was (cataract → blindness). The flow in metabolic pathway, Axon guidance, of cataract progress through the metabolic pathway, MAPK signaling pathway, of blindness. Thus, [14] extracts that cataract causes blindness, which agree with the result of the proposed method.

### Result comparison with previous study

The proposed method for causal disease network is compared with dRiskKB of [22]. dRiskKB considered causality strength using semi-supervised iterative pattern learning approach based on sentence frequency. Here, higher sentence frequencies in corpus that indicate disease causality implies higher causality strength. However, dRiskKB did not incorporate the concept of “causality term strength” which refers to the strength of causal connotation. By considering causality term strength, it is possible to take into account for implicit meaning of the terms, while the terms are treated with equal connotation if not considered. Comparison has been done in two aspects of coverage and quality. The coverage comparison aims to validate quantitative aspects such as which method discovers more causalities among diseases, whereas quality comparison aims to verify which method finds more relevant causalities. Note that, for convenience, we hereafter use $\alpha DCFC$ to indicate our method.

#### Coverage comparison

**Fig. 5:**
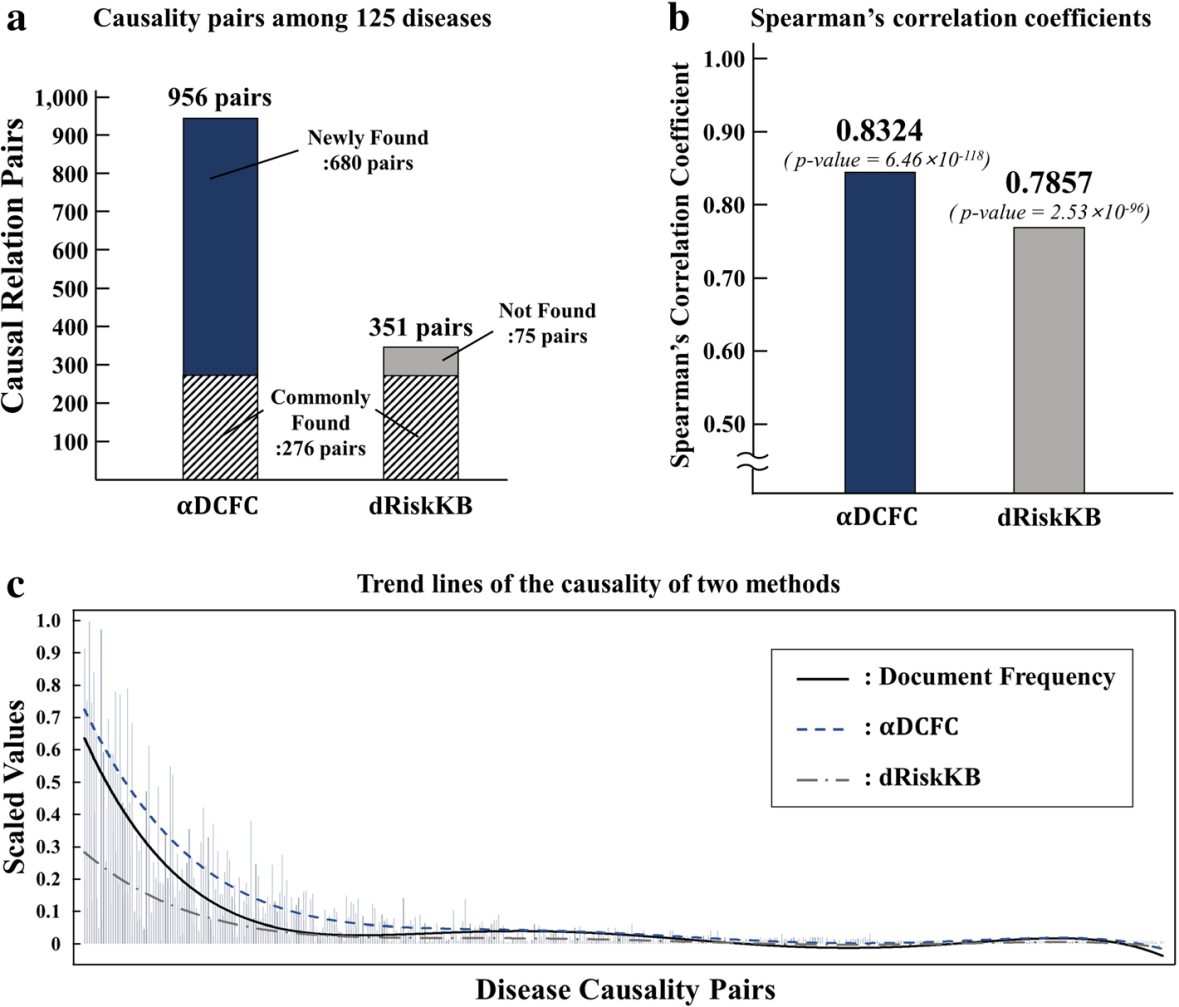
Experimental Results: **a** For 125 diseases, $\alpha DCFC$ extracted 2.7 times more causal relationship compared with dRiskKB, and covered 79% of the existing causal relationships. **b** When the ranks of the causality strength are compared, the proposed method has a higher rank correlation coefficient with the document frequency than that of dRiskKB; the document frequency represents the causal relationships. **c** Trend lines of the causality strength of two methods show that $\alpha DCFC$ is more similar to the document frequency than dRiskKB

The number of common diseases that the two methods share was 125. For these diseases, dRiskKB extracted 351 causal relationships, whereas $\alpha DCFC$ found 956 causal relationships. Specifically, 276 causal relationships found by $\alpha DCFC$ were common with dRiskKB. Further, $\alpha DCFC$ found 680 more causalities [see Fig. 5a]. The experimental results suggest that the proposed method more efficiently extracted disease causalities than the existing method. Technically speaking, it is superior to dRiskKB by covering 2.7 times larger number of causalities.

#### Quality comparison

Identifying the relevance of disease causalities is indeed difficult irrespective of what $\alpha DCFC$ or dRiskKB finds. One of the methods to solve this difficulty may be to compare the causality strength with the document frequency, which could be justified because it raises more confidence on the causality if many documents have reported on the causal relationship of a certain pair of diseases. The comparison is fair to $\alpha DCFC$ and dRiskKB because both are frequency-based methods.

For the 43,248 documents of the 6,617,833 PubMed abstracts, 454 pairs of 125 diseases were shared by $\alpha DCFC$ and dRiskKB. Then, for these 454 causalities, the values of the causality strength obtained by $\alpha DCFC$ were calculated, and rank correlation with the document frequency was calculated. This procedure was similarly applied to dRiskKB.

Figure 5b shows the Spearman’s rank correlation coefficient. $\alpha DCFC$ shows correlation of 0.83, whereas dRiskKB shows correlation of 0.79. The results show that the causalities obtained by the proposed method provide more relevance with respect to the document frequency. Figure 5c visually depicts the results of quality comparison for the causality strength values. Each bar represents scaled values (between zero and one) of document frequency and causality strengths for each pair. The trend lines are calculated by polynomial curve fitting. In the figure, the trend line of $\alpha DCFC$ shows closer association with the document frequency than that of dRiskKB. Table 5 lists the top 10 disease pairs sorted by document frequency, which shows the ranks of the disease pairs. We can see a closed association with the document frequency from the causality strength of $\alpha DCFC$.

**Table 5:** Comparison of causality strengths and ranks by the top-10 ranked pairs in the document frequency: The document frequency and causality strengths of both $\alpha DCFC$ and dRiskKB have different range of values; so each value is scaled between zero and one

Disease Causality Pairs			Document Frequency		$\alpha DCFC$		dRiskKB	
			Frequency	Rank	Causality Strength	Rank	Causality Strength	Rank
Hepatitis C	→	Liver Diseases	1.00	1	0.70	3	0.20	12
Hepatitis B	→	Carcinoma, Hepatocellular	0.90	2	0.39	9	0.11	33
Aneurysm	→	Hemorrhage	0.76	3	1.00	1	1.00	1
Hepatitis C	→	Carcinoma, Hepatocellular	0.72	4	0.49	5	0.13	26
Thrombosis	→	Infarction	0.63	5	0.53	4	0.41	4
Hepatitis B	→	Liver Diseases	0.61	6	0.28	12	0.14	22
Rectal Neoplasms	→	Adenoma	0.61	6	0.01	164	0.05	59
Glaucoma	→	Blindness	0.59	8	0.89	2	0.08	45
Infarction	→	Heart Failure	0.47	9	0.23	14	0.22	11
Hemorrhage	→	Stroke	0.45	10	0.07	44	0.42	3

## Conclusions

In this paper, we have proposed two methods that extract the causalities between diseases from biomedical literature, namely, *lexicon-based causality term strength* and *frequency-based causality strength*. The former provides the causal strength of a variety of causality terms based on lexicon analysis, whereas the latter determines the direction and strength of causality based on DCF. The results were illustrated as a disease network whose edges now have directions showing prior and posterior diseases.

The novelty of the present research is described by the following aspects. First, causal disease network incorporates relevant biological or clinical reports through text mining. In effect, this process circumvents the limitations of time and cost in applying all possible causalities in biological experiments. To extract prior–posterior information from 6,617,833 abstracts, we proposed an efficient text mining model. Second, in the methodological aspect, defining the concepts of causality term strength based on lexical semantics and causality frequency-based biomedical literature is a more advanced text mining technique compared with existing models as the proposed method more finely reflects the prior–posterior disease information. When the proposed method was compared with previous research, namely, dRiskKB [22], the proposed method showed outperforming results; it determined 2.7 times more causalities and showed higher correlation with associated diseases than the existing method.

This research can exploit more extended research. First, in the present research, we only used approximately 6 million literature of PubMed. However, if we use more than 20 million of the whole literature, we would be able to provide more generalized results that cover existing documents. Second, we only applied the method to 195 diseases in the current research because of time limitation. If we expand this method to all listed diseases in MeSH, which covers approximately 4663 diseases, we can further extend the disease causalities for wider range of diseases. Third, decades may be needed to verify our experimental results more thoroughly— observing patients for years to see if they actually experience the projected disease causality. The other option for validation may be by comparing our results from text literature with those obtained from biological-level experiments, which will be our next research.

## Additional file

Additional file 1: Table S1. 195 Diseases. **Table S2**. Disease causality pairs (1011 pairs). **Figure S1**. Causal disease network of 149 diseases. (PDF 1173 kb)
