# Supplementary material for: Disease causality extraction based on lexical semantics and document-clause frequency from biomedical literature
Source: BMC Med Inform Decis Mak. 2017 May 18;17(Suppl 1):53. doi: 10.1186/s12911-017-0448-y (PMC5444051; doi:10.1186/s12911-017-0448-y)
Supplement: Additional file 1: Table S1. — 195 Diseases. Table S2. Disease causality pairs (1011 pairs). Figure S1. Causal disease network of 149 diseases. (PDF 1173 kb) [file 12911_2017_448_MOESM1_ESM.pdf]

Additional file

Disease Causality Extraction based on Lexical Semantics and Document-Clause Frequency from Biomedical Literature

Dong-gi Lee and Hyunjung Shin*
Department of Industrial Engineering, Ajou University,
206 Worldcup-ro, Yeongtong-gu, Suwon 16499, South Korea

< Supplements >

- **Table S1: 195 Diseases**
- **Table S2: Disease Causality Pairs**
- **Figure S1: Causal Disease Network of 149 diseases**

*Corresponding author: Hyunjung (Helen) Shin, shin@ajou.ac.kr

Table S1. 195 Diseases

1	Abscess	66	Fibromuscular Dysplasia	131	Neoplasm Recurrence, Local
2	Achondroplasia	67	Fistula	132	Neoplasm Regression, Spontaneous
3	Acrospiroma	68	Focal Nodular Hyperplasia	133	Neoplasms by Site
4	ACTH Syndrome, Ectopic	69	Gastroenteritis	134	Nephrotic Syndrome
5	ACTH-Secreting Pituitary Adenoma	70	Gastroesophageal Reflux	135	Neurogenic Bowel
6	Adenocarcinoma, Clear Cell	71	Gingival Hyperplasia	136	Nonodontogenic Cysts
7	Adenocarcinoma, Follicular	72	Gingivitis, Necrotizing Ulcerative	137	Onchocerciasis
8	Adenocarcinoma, Papillary	73	Glaucoma	138	Osteoarthritis
9	Adenoma	74	Graves Disease	139	Osteoarthropathy, Secondary Hypertrophic
10	Adrenocortical Carcinoma	75	Haemophilus Infections	140	Osteomalacia
11	alpha 1-Antitrypsin Deficiency	76	Heart Failure	141	Osteopoikilosis
12	Amblyopia	77	Hemiplegia	142	Osteosclerosis
13	Anaplasmataceae Infections	78	Hemorrhage	143	Pancreatitis, Graft
14	Anaplasmosis	79	Hepatitis B	144	Panniculitis, Peritoneal
15	Aneurysm	80	Hepatitis C	145	Paraplegia
16	Angina Pectoris	81	Hepatitis, Alcoholic	146	Peptic Ulcer
17	Anisometropia	82	Hepatitis, Animal	147	Pericardial Effusion
18	Anovulation	83	Herpes Simplex	148	Peripheral Arterial Disease
19	Anus Diseases	84	Herpes Zoster	149	Peritonitis
20	Appendicitis	85	Hydrocephalus	150	Pituitary Neoplasms
21	Ascariasis	86	Hydronephrosis	151	Placenta Accreta
22	Bacterial Infections and Mycoses	87	Hypercalcemia	152	Placenta Previa
23	Biliary Atresia	88	Hyperinsulinism	153	Pleural Effusion
24	Blindness	89	Hyperparathyroidism	154	Pleuropneumonia, Contagious
25	Bone Diseases	90	Hypersplenism	155	Pneumatosis Cystoides Intestinalis
26	Bone Diseases, Endocrine	91	Hyperthyroidism	156	Pneumonia
27	Brain Neoplasms	92	Hypocalcemia	157	Postgastrectomy Syndromes
28	Bronchial Neoplasms	93	Hypoglycemia	158	Pregnancy Complications, Neoplastic
29	Carcinoma, Acinar Cell	94	Hypoparathyroidism	159	Protein-Losing Enteropathies
30	Carcinoma, Hepatocellular	95	Hypotension	160	Pruritus Ani
31	Cardiac Tamponade	96	Infarction	161	Pseudomonas Infections
32	Cataract	97	Infertility	162	Pyloric Stenosis, Hypertrophic
33	Cerebral Amyloid Angiopathy	98	Insulinoma	163	Q Fever
34	Cerebral Infarction	99	Intermittent Claudication	164	Rectal Neoplasms
35	Cerebral Ventricle Neoplasms	100	Intestinal Obstruction	165	Retinal Vein Occlusion
36	Chalazion	101	Intestinal Perforation	166	Rhabdoviridae Infections
37	Chancroid	102	Intestinal Pseudo-Obstruction	167	Rickets
38	Chest Pain	103	Intracranial Aneurysm	168	Salivary Gland Fistula
39	Chronic Pain	104	Intussusception	169	Scheuermann Disease
40	Cleidocranial Dysplasia	105	Irritable Bowel Syndrome	170	Sepsis
41	Cockayne Syndrome	106	Ischemia	171	Sigmoid Diseases
42	Congenital Hypothyroidism	107	Kearns-Sayre Syndrome	172	Sinusitis
43	Coronary Artery Disease	108	Keratoconjunctivitis, Infectious	173	Spinal Cord Ischemia
44	Coronary Occlusion	109	Laron Syndrome	174	Spondylitis
45	Dehydration	110	Liver Cirrhosis	175	Stomach Rupture
46	Dental Occlusion, Traumatic	111	Liver Diseases	176	Strabismus
47	Diabetic Retinopathy	112	Loeys-Dietz Syndrome	177	Stroke
48	Diarrhea	113	Lupus Nephritis	178	Subarachnoid Hemorrhage
49	Discitis	114	Lymphoma, Large B-Cell, Diffuse	179	Sweat Gland Neoplasms
50	Diverticulitis	115	Macular Edema	180	Syndactyly
51	Diverticulitis, Colonic	116	Malignant Carcinoid Syndrome	181	Thrombocytopenia
52	Drug-Induced Liver Injury	117	Mediastinitis	182	Thrombocytosis
53	Drug-Induced Liver Injury, Chronic	118	Melanoma, Amelanotic	183	Thrombosis
54	Dry Socket	119	Melorheostosis	184	Thyroid Dysgenesis
55	Ectromelia	120	Meningitis	185	Toxoplasmosis, Animal
56	Encephalitis	121	Meningitis, Bacterial	186	Trachoma
57	Enchondromatosis	122	Mikulicz' Disease	187	Tuberculosis, Cutaneous
58	End Stage Liver Disease	123	Mouth Neoplasms	188	Tuberculosis, Urogenital
59	Endometriosis	124	Multiple Myeloma	189	Typhoid Fever
60	Esophageal Perforation	125	Mumps	190	Ureteral Obstruction
61	Esophagitis	126	Muscular Dystrophy, Facioscapulohumeral	191	Vascular Neoplasms
62	Eyelid Neoplasms	127	Myeloproliferative Disorders	192	Vitamin D Deficiency
63	Facial Hemiatrophy	128	Myocardial Infarction	193	Vitamin K Deficiency
64	Fecal Impaction	129	Myocardial Ischemia	194	Weill-Marchesani Syndrome
65	Fecal Incontinence	130	Neisseriaceae Infections	195	Zellweger Syndrome

Table S2. Disease Causality Pairs (1,011 pairs)

Prior Disease	Posterior Disease	$\alpha DCFC$	Prior Disease	Posterior Disease	$\alpha DCFC$
Aneurysm	Hemorrhage	140.38	Diarrhea	Hypotension	0.30
Glaucoma	Blindness	125.23	Diverticulitis	Irritable Bowel Syndrome	0.30
Hepatitis C	Liver Diseases	97.58	Encephalitis	Thrombocytopenia	0.30
Thrombosis	Infarction	73.78	Endometriosis	Intestinal Obstruction	0.30
Hepatitis C	Carcinoma, Hepatocellular	68.21	Endometriosis	Hydronephrosis	0.30
Cataract	Blindness	62.04	Endometriosis	Pleural Effusion	0.30
Pneumonia	Meningitis	59.73	Esophageal Perforation	Fistula	0.30
Aneurysm	Subarachnoid Hemorrhage	58.17	Esophageal Perforation	Hemorrhage	0.30
Hepatitis B	Carcinoma, Hepatocellular	54.74	Esophagitis	Hemorrhage	0.30
Thrombosis	Myocardial Infarction	45.48	Fibromuscular Dysplasia	Fistula	0.30
Diabetic Retinopathy	Blindness	43.51	Fistula	Meningitis, Bacterial	0.30
Hepatitis B	Liver Diseases	39.52	Fistula	Endometriosis	0.30
Adenoma	Hyperparathyroidism	34.86	Fistula	Graves Disease	0.30
Infarction	Heart Failure	32.70	Fistula	Liver Cirrhosis	0.30
Hepatitis C	Liver Cirrhosis	32.29	Gastroenteritis	Typhoid Fever	0.30
Infarction	Stroke	32.20	Gastroenteritis	Appendicitis	0.30
Retinal Vein Occlusion	Macular Edema	28.51	Gastroenteritis	Blindness	0.30
Myocardial Infarction	Heart Failure	26.51	Gastroenteritis	Hemiplegia	0.30
Graves Disease	Hyperthyroidism	26.28	Gastroesophageal Reflux	Esophageal Perforation	0.30
Thrombosis	Ischemia	23.23	Gastroesophageal Reflux	Fistula	0.30
Endometriosis	Infertility	22.13	Gastroesophageal Reflux	Hypercalcemia	0.30
Thrombosis	Stroke	19.59	Graves Disease	Glaucoma	0.30
Ischemia	Infarction	19.55	Graves Disease	Heart Failure	0.30
Adenoma	Hyperthyroidism	18.58	Graves Disease	Hemorrhage	0.30
Pneumonia	Sepsis	18.56	Graves Disease	Hepatitis C	0.30
Drug-Induced Liver Injury	Liver Diseases	16.63	Graves Disease	Stroke	0.30
Pituitary Neoplasms	Hyperthyroidism	16.00	Heart Failure	Kearns-Sayre Syndrome	0.30
Trachoma	Blindness	15.33	Heart Failure	Protein-Losing Enteropathies	0.30
Hyperparathyroidism	Hypercalcemia	14.51	Heart Failure	Spondylitis	0.30
Fistula	Hemorrhage	13.55	Heart Failure	Abscess	0.30
Hemorrhage	Infarction	13.39	Hemiplegia	Blindness	0.30
Intracranial Aneurysm	Hemorrhage	13.07	Hemiplegia	Pneumonia	0.30
Liver Diseases	Carcinoma, Hepatocellular	12.60	Hemorrhage	End Stage Liver Disease	0.30
Chest Pain	Infarction	12.28	Hemorrhage	Irritable Bowel Syndrome	0.30
Angina Pectoris	Infarction	12.25	Hemorrhage	Meningitis, Bacterial	0.30
Angina Pectoris	Myocardial Infarction	11.95	Hemorrhage	Pancreatitis, Graft	0.30
Intracranial Aneurysm	Subarachnoid Hemorrhage	11.03	Hemorrhage	Chest Pain	0.30
Hepatitis B	Liver Cirrhosis	10.96	Hemorrhage	Encephalitis	0.30
Chest Pain	Myocardial Infarction	10.83	Hemorrhage	Herpes Simplex	0.30
Pneumonia	Abscess	10.48	Hemorrhage	Hypocalcemia	0.30
Intussusception	Intestinal Obstruction	10.39	Hemorrhage	Mediastinitis	0.30
Abscess	Fistula	9.81	Hemorrhage	Myocardial Ischemia	0.30
Myocardial Ischemia	Heart Failure	9.61	Hepatitis B	Hepatitis, Animal	0.30
Hemorrhage	Stroke	9.31	Hepatitis B	Osteomalacia	0.30
Ischemia	Myocardial Infarction	9.25	Hepatitis B	Thrombosis	0.30
Hemorrhage	Myocardial Infarction	9.13	Hepatitis C	Ischemia	0.30
Herpes Simplex	Encephalitis	9.04	Herpes Simplex	Cockayne Syndrome	0.30
Cerebral Amyloid Angiopathy	Hemorrhage	8.86	Herpes Zoster	Adenoma	0.30
Coronary Artery Disease	Heart Failure	8.77	Herpes Zoster	Intestinal Pseudo-Obstruction	0.30
Aneurysm	Infarction	8.32	Herpes Zoster	Q Fever	0.30
Hemorrhage	Hypotension	7.91	Herpes Zoster	Pneumonia	0.30
Pituitary Neoplasms	Stroke	7.32	Hydronephrosis	Carcinoma, Hepatocellular	0.30
Hypotension	Ischemia	7.26	Hydronephrosis	Discitis	0.30
Gastroesophageal Reflux	Esophagitis	7.13	Hydronephrosis	Ischemia	0.30
Thrombocytopenia	Hemorrhage	7.07	Hypercalcemia	Peritonitis	0.30
Pituitary Neoplasms	Adenoma	7.04	Hyperinsulinism	Adenoma	0.30
Pneumonia	Meningitis, Bacterial	6.81	Hyperparathyroidism	Osteomalacia	0.30
Coronary Artery Disease	Infarction	6.54	Hyperparathyroidism	Stroke	0.30
Adenoma	Stroke	6.44	Hyperthyroidism	Bone Diseases	0.30
Aneurysm	Fistula	6.35	Hyperthyroidism	Thyroid Dysgenesis	0.30
Peptic Ulcer	Hemorrhage	6.26	Hypocalcemia	Hypotension	0.30
Liver Diseases	Liver Cirrhosis	6.23	Hypoglycemia	Myocardial Ischemia	0.30
Trachoma	Infertility	5.67	Hypoparathyroidism	Spondylitis	0.30
Ischemia	Myocardial Ischemia	5.57	Hypotension	Adenoma	0.30
Aneurysm	Thrombosis	5.56	Hypotension	Pituitary Neoplasms	0.30
Hyperinsulinism	Hypoglycemia	5.37	Hypotension	Cerebral Infarction	0.30
Fistula	Heart Failure	5.27	Hypotension	Liver Diseases	0.30
Cataract	Glaucoma	5.16	Hypotension	Myocardial Ischemia	0.30
Adenoma	Hypercalcemia	5.15	Hypotension	Stroke	0.30
Liver Cirrhosis	Carcinoma, Hepatocellular	5.15	Infarction	Irritable Bowel Syndrome	0.30
Hemorrhage	Hydrocephalus	5.07	Infarction	Hemiplegia	0.30
Ischemia	Paraplegia	4.97	Infarction	Hydronephrosis	0.30
Meningitis	Hydrocephalus	4.87	Infarction	Hypercalcemia	0.30
Myocardial Ischemia	Infarction	4.80	Infertility	Hydrocephalus	0.30
Ischemia	Heart Failure	4.80	Infertility	Hyperinsulinism	0.30
Coronary Artery Disease	Myocardial Infarction	4.74	Infertility	Hyperthyroidism	0.30
Diabetic Retinopathy	Macular Edema	4.73	Intracranial Aneurysm	Cerebral Infarction	0.30
Diarrhea	Dehydration	4.69	Intracranial Aneurysm	Infarction	0.30
Aneurysm	Stroke	4.69	Intracranial Aneurysm	Sepsis	0.30
Peritonitis	Intestinal Obstruction	4.64	Intussusception	Intestinal Pseudo-Obstruction	0.30
Ischemia	Chest Pain	4.57	Intussusception	Hemorrhage	0.30
Thyroid Dysgenesis	Congenital Hypothyroidism	4.53	Ischemia	Hemiplegia	0.30
Sepsis	Thrombosis	4.47	Ischemia	Strabismus	0.30
Myocardial Ischemia	Myocardial Infarction	4.27	Ischemia	Ureteral Obstruction	0.30
Hepatitis B	End Stage Liver Disease	4.26	Liver Cirrhosis	Meningitis, Bacterial	0.30

Vitamin D Deficiency	Rickets	4.24	Liver Cirrhosis	Esophagitis	0.30
Liver Diseases	Hepatitis, Alcoholic	4.10	Liver Cirrhosis	Heart Failure	0.30
Trachoma	Pneumonia	4.02	Liver Cirrhosis	Hypoglycemia	0.30
Aneurysm	Heart Failure	3.97	Liver Cirrhosis	Meningitis	0.30
Thrombosis	Thrombocytopenia	3.91	Liver Cirrhosis	Peptic Ulcer	0.30
Coronary Artery Disease	Angina Pectoris	3.89	Liver Cirrhosis	Thrombosis	0.30
Aneurysm	Myocardial Infarction	3.74	Liver Diseases	Infarction	0.30
Heart Failure	Stroke	3.67	Liver Diseases	Myocardial Infarction	0.30
Diverticulitis	Abscess	3.56	Lupus Nephritis	Hemorrhage	0.30
Hemorrhage	Ischemia	3.52	Meningitis	Brain Neoplasms	0.30
Anisometropia	Amblyopia	3.51	Meningitis	Cardiac Tamponade	0.30
Infarction	Myocardial Infarction	3.45	Meningitis	Gastroenteritis	0.30
Herpes Zoster	Encephalitis	3.38	Meningitis	Hydronephrosis	0.30
Fistula	Sepsis	3.36	Meningitis	Ischemia	0.30
Osteoarthritis	Chronic Pain	3.25	Meningitis	Liver Diseases	0.30
Thrombosis	Fistula	3.20	Meningitis	Strabismus	0.30
Thrombosis	Hemorrhage	3.17	Myeloproliferative Disorders	Thrombosis	0.30
Diverticulitis	Fistula	3.16	Myocardial Infarction	Cardiac Tamponade	0.30
Hepatitis B	Hepatitis C	3.02	Myocardial Infarction	Hydronephrosis	0.30
Diverticulitis	Peritonitis	2.99	Myocardial Infarction	Hypercalcemia	0.30
Hemorrhage	Liver Diseases	2.97	Myocardial Infarction	Paraplegia	0.30
Peritonitis	Sepsis	2.93	Myocardial Infarction	Subarachnoid Hemorrhage	0.30
Adenoma	Intussusception	2.86	Myocardial Ischemia	Intestinal Obstruction	0.30
Intracranial Aneurysm	Stroke	2.84	Nephrotic Syndrome	Hypercalcemia	0.30
Myocardial Ischemia	Chest Pain	2.80	Nephrotic Syndrome	Liver Cirrhosis	0.30
Ischemia	Cerebral Infarction	2.75	Osteomalacia	Bone Diseases	0.30
Drug-Induced Liver Injury	Hepatitis B	2.69	Paraplegia	Insulinoma	0.30
Placenta Previa	Hemorrhage	2.66	Paraplegia	Hemiplegia	0.30
Thrombosis	Heart Failure	2.63	Peptic Ulcer	Carcinoma, Hepatocellular	0.30
Vitamin K Deficiency	Hemorrhage	2.57	Peptic Ulcer	Appendicitis	0.30
Stroke	Hemiplegia	2.53	Peptic Ulcer	Ischemia	0.30
Multiple Myeloma	Bone Diseases	2.48	Peptic Ulcer	Stroke	0.30
Adenoma	Hemorrhage	2.47	Peritonitis	Carcinoma, Hepatocellular	0.30
Vitamin D Deficiency	Hyperparathyroidism	2.45	Peritonitis	Typhoid Fever	0.30
Hypoparathyroidism	Hypocalcemia	2.45	Peritonitis	Infertility	0.30
Mumps	Meningitis	2.38	Peritonitis	Liver Diseases	0.30
Aneurysm	Intracranial Aneurysm	2.38	Peritonitis	Ureteral Obstruction	0.30
Sepsis	Abscess	2.37	Pleural Effusion	Cardiac Tamponade	0.30
Aneurysm	Hydrocephalus	2.36	Pleural Effusion	Hypoglycemia	0.30
Aneurysm	Ischemia	2.35	Pleural Effusion	Hypotension	0.30
Thrombocytosis	Thrombosis	2.31	Pleural Effusion	Peritonitis	0.30
Intestinal Perforation	Peritonitis	2.26	Pneumonia	Pleuropneumonia, Contagious	0.30
Myeloproliferative Disorders	Thrombocytosis	2.26	Pneumonia	Cerebral Infarction	0.30
Multiple Myeloma	Hypercalcemia	2.17	Pneumonia	Myocardial Infarction	0.30
Strabismus	Amblyopia	2.16	Retinal Vein Occlusion	Amblyopia	0.30
Fistula	Ischemia	2.08	Retinal Vein Occlusion	Stroke	0.30
Hyperparathyroidism	Bone Diseases	2.06	Rickets	Hypocalcemia	0.30
Anovulation	Infertility	1.96	Sepsis	Discitis	0.30
Retinal Vein Occlusion	Blindness	1.93	Sepsis	Dehydration	0.30
Endometriosis	Hemorrhage	1.92	Sepsis	Esophageal Perforation	0.30
Appendicitis	Peritonitis	1.89	Sepsis	Heart Failure	0.30
Hydronephrosis	Sepsis	1.88	Sinusitis	Aneurysm	0.30
Paraplegia	Aneurysm	1.87	Stroke	Kearns-Sayre Syndrome	0.30
Insulinoma	Hypoglycemia	1.86	Stroke	Abscess	0.30
Herpes Simplex	Meningitis	1.86	Stroke	Intermittent Claudication	0.30
Meningitis	Meningitis, Bacterial	1.86	Stroke	Nephrotic Syndrome	0.30
Thrombosis	Cerebral Infarction	1.81	Subarachnoid Hemorrhage	Adenoma	0.30
Gastroenteritis	Irritable Bowel Syndrome	1.81	Subarachnoid Hemorrhage	Pituitary Neoplasms	0.30
Herpes Zoster	Stroke	1.81	Subarachnoid Hemorrhage	Cerebral Infarction	0.30
Pericardial Effusion	Heart Failure	1.81	Subarachnoid Hemorrhage	Dehydration	0.30
Spinal Cord Ischemia	Paraplegia	1.76	Subarachnoid Hemorrhage	Sepsis	0.30
Fecal Impaction	Intestinal Obstruction	1.76	Thrombocytopenia	Adenoma	0.30
Pneumonia	Peritonitis	1.76	Thrombocytopenia	Bone Diseases	0.30
Sepsis	Thrombocytopenia	1.73	Thrombocytopenia	Intestinal Pseudo-Obstruction	0.30
Aneurysm	Cardiac Tamponade	1.71	Thrombocytopenia	Multiple Myeloma	0.30
Hypocalcemia	Heart Failure	1.71	Thrombocytopenia	Hypercalcemia	0.30
Coronary Occlusion	Myocardial Ischemia	1.66	Thrombocytopenia	Infertility	0.30
Gastroenteritis	Diarrhea	1.66	Thrombocytosis	Coronary Artery Disease	0.30
Coronary Occlusion	Ischemia	1.58	Thrombocytosis	Nephrotic Syndrome	0.30
Drug-Induced Liver Injury	Hepatitis C	1.57	Thrombosis	Brain Neoplasms	0.30
Aneurysm	Ureteral Obstruction	1.56	Thrombosis	Coronary Occlusion	0.30
Hypoparathyroidism	Heart Failure	1.56	Thrombosis	Hemiplegia	0.30
Infarction	Intestinal Obstruction	1.56	Thrombosis	Herpes Zoster	0.30
Hemorrhage	Liver Cirrhosis	1.55	Thrombosis	Retinal Vein Occlusion	0.30
Hepatitis, Alcoholic	Carcinoma, Hepatocellular	1.48	Thrombosis	Spinal Cord Ischemia	0.30
Hemorrhage	Meningitis	1.48	Thyroid Dysgenesis	Adenoma	0.30
Ischemia	Glaucoma	1.47	Vitamin D Deficiency	Chronic Pain	0.30
Diverticulitis	Hemorrhage	1.46	Vitamin D Deficiency	Peripheral Arterial Disease	0.30
Rectal Neoplasms	Adenoma	1.43	Subarachnoid Hemorrhage	Ischemia	0.30
Stroke	Ischemia	1.41	Heart Failure	Pneumonia	0.29
Rectal Neoplasms	Diarrhea	1.41	Pneumonia	Fistula	0.29
Vitamin D Deficiency	Hypocalcemia	1.36	Stroke	Meningitis	0.28
Heart Failure	Pleural Effusion	1.35	Adenocarcinoma, Clear Cell	Endometriosis	0.25
Drug-Induced Liver Injury	Carcinoma, Hepatocellular	1.35	Hepatitis C	Hemorrhage	0.25
Thrombocytopenia	Diarrhea	1.32	Infarction	Adenoma	0.25
Pericardial Effusion	Cardiac Tamponade	1.30	Hemorrhage	Abscess	0.25
Myocardial Infarction	Stroke	1.29	Hemorrhage	Pneumonia	0.25
Hepatitis C	End Stage Liver Disease	1.28	Hepatitis B	Hemorrhage	0.24

Adenoma	Infertility	1.26
Pituitary Neoplasms	Infertility	1.26
Spondylitis	Aneurysm	1.26
Aneurysm	Cerebral Infarction	1.26
Herpes Simplex	Esophagitis	1.26
Hydrocephalus	Ischemia	1.26
Hyperparathyroidism	Hypoparathyroidism	1.26
Vitamin D Deficiency	Osteomalacia	1.25
Aneurysm	Macular Edema	1.24
Fibromuscular Dysplasia	Aneurysm	1.20
Myocardial Ischemia	Angina Pectoris	1.18
Glaucoma	Diabetic Retinopathy	1.17
Peripheral Arterial Disease	Intermittent Claudication	1.15
Infarction	Pituitary Neoplasms	1.13
Retinal Vein Occlusion	Hemorrhage	1.13
Esophageal Perforation	Mediastinitis	1.08
Hemorrhage	Pituitary Neoplasms	1.08
Carcinoma, Hepatocellular	Rectal Neoplasms	1.07
Hypotension	Infarction	1.06
Hemorrhage	Cardiac Tamponade	1.05
Liver Cirrhosis	Hypersplenism	1.05
Myocardial Ischemia	Stroke	1.05
Thrombosis	Peripheral Arterial Disease	1.05
Hemorrhage	Cerebral Infarction	1.04
Cerebral Infarction	Stroke	1.03
Hemorrhage	Subarachnoid Hemorrhage	1.02
Rickets	Osteomalacia	1.02
Pneumonia	Encephalitis	1.00
Pituitary Neoplasms	Meningitis	0.98
Diverticulitis	Intestinal Obstruction	0.98
Typhoid Fever	Hemorrhage	0.95
Cerebral Amyloid Angiopathy	Ischemia	0.95
Hemorrhage	Focal Nodular Hyperplasia	0.95
Ischemia	Angina Pectoris	0.95
Meningitis	Abscess	0.95
Stroke	Dehydration	0.95
Thrombocytosis	Infarction	0.95
Thrombocytosis	Myocardial Infarction	0.95
Herpes Zoster	Infertility	0.94
Retinal Vein Occlusion	Glaucoma	0.94
Mediastinitis	Abscess	0.94
Coronary Artery Disease	Aneurysm	0.90
Coronary Artery Disease	Stroke	0.90
Fibromuscular Dysplasia	Infarction	0.90
Fistula	Myocardial Ischemia	0.90
Hypocalcemia	Hyperparathyroidism	0.90
Sepsis	Meningitis	0.90
Thrombosis	Intracranial Aneurysm	0.90
Drug-Induced Liver Injury	Hepatitis, Alcoholic	0.90
Thrombocytopenia	Heart Failure	0.90
Hypotension	Sepsis	0.88
Pneumonia	Pleural Effusion	0.88
Adenoma	Carcinoma, Hepatocellular	0.88
Coronary Artery Disease	Ischemia	0.87
Hemorrhage	Carcinoma, Hepatocellular	0.85
Ischemia	Sepsis	0.84
Brain Neoplasms	Hemorrhage	0.83
Fistula	Meningitis	0.83
Herpes Simplex	Infertility	0.83
Hydrocephalus	Pituitary Neoplasms	0.83
Hypotension	Thrombosis	0.83
Liver Diseases	Thrombocytopenia	0.83
Pituitary Neoplasms	Hyperparathyroidism	0.80
Appendicitis	Sepsis	0.78
Intracranial Aneurysm	Hydrocephalus	0.78
Coronary Occlusion	Myocardial Infarction	0.75
Peptic Ulcer	Fistula	0.75
Stroke	Blindness	0.75
Liver Cirrhosis	Thrombocytopenia	0.75
Adenoma	Multiple Myeloma	0.73
Infarction	Sepsis	0.73
Typhoid Fever	Diarrhea	0.72
Pleural Effusion	Pericardial Effusion	0.72
Adenoma	Diverticulitis	0.68
Adenoma	Meningitis	0.68
Aneurysm	Abscess	0.68
Aneurysm	Myocardial Ischemia	0.68
Myocardial Infarction	Fistula	0.68
Infarction	Meningitis	0.68
Gastroesophageal Reflux	Pneumonia	0.66
End Stage Liver Disease	Liver Diseases	0.65
Hypotension	Paraplegia	0.65
Intestinal Perforation	Sepsis	0.65
Pneumonia	Sinusitis	0.65
Fistula	Appendicitis	0.63
Gastroenteritis	Dehydration	0.63
Bone Diseases	Hypercalcemia	0.61
Hemorrhage	Sepsis	0.61

Adenocarcinoma, Clear Cell	Adenoma	0.23
Adenocarcinoma, Papillary	Rectal Neoplasms	0.23
Adenoma	ACTH-Secreting Pituitary Adenoma	0.23
Adenoma	Hepatitis, Alcoholic	0.23
Adenoma	Melanoma, Amelanotic	0.23
Adenoma	Glaucoma	0.23
Adrenocortical Carcinoma	Carcinoma, Hepatocellular	0.23
Biliary Atresia	Intussusception	0.23
Bone Diseases	Hemorrhage	0.23
Carcinoma, Acinar Cell	Adenocarcinoma, Papillary	0.23
Carcinoma, Hepatocellular	Herpes Simplex	0.23
Cerebral Ventricle Neoplasms	Hydrocephalus	0.23
Chalazion	Irritable Bowel Syndrome	0.23
Diverticulitis, Colonic	Intestinal Obstruction	0.23
Diverticulitis, Colonic	Abscess	0.23
Diverticulitis, Colonic	Fistula	0.23
End Stage Liver Disease	Ischemia	0.23
Focal Nodular Hyperplasia	Liver Diseases	0.23
Insulinoma	Pituitary Neoplasms	0.23
Insulinoma	Hyperparathyroidism	0.23
Intestinal Obstruction	Abscess	0.23
Intestinal Obstruction	Aneurysm	0.23
Intestinal Pseudo-Obstruction	Sepsis	0.23
Meningitis, Bacterial	Stroke	0.23
Multiple Myeloma	Carcinoma, Hepatocellular	0.23
Multiple Myeloma	Spondylitis	0.23
Multiple Myeloma	Hypocalcemia	0.23
Mumps	Diarrhea	0.23
Pituitary Neoplasms	Glaucoma	0.23
Q Fever	Pneumonia	0.23
Rectal Neoplasms	Adrenocortical Carcinoma	0.23
Rectal Neoplasms	Hypotension	0.23
Rectal Neoplasms	Thrombocytosis	0.23
Rectal Neoplasms	Thrombosis	0.23
Spondylitis	Paraplegia	0.23
Typhoid Fever	Mumps	0.23
Abscess	Focal Nodular Hyperplasia	0.23
Abscess	Spondylitis	0.23
Abscess	Paraplegia	0.23
Aneurysm	Pneumonia	0.23
Appendicitis	Intussusception	0.23
Blindness	Strabismus	0.23
Cardiac Tamponade	Heart Failure	0.23
Cardiac Tamponade	Ischemia	0.23
Cataract	Abscess	0.23
Cataract	Hyperthyroidism	0.23
Cerebral Infarction	Herpes Zoster	0.23
Chest Pain	Hypotension	0.23
Chest Pain	Pneumonia	0.23
Chronic Pain	Encephalitis	0.23
Chronic Pain	Paraplegia	0.23
Congenital Hypothyroidism	Pituitary Neoplasms	0.23
Coronary Artery Disease	Intermittent Claudication	0.23
Diabetic Retinopathy	Hemorrhage	0.23
Diarrhea	Cataract	0.23
Diarrhea	Thrombocytosis	0.23
Encephalitis	Diarrhea	0.23
Endometriosis	Appendicitis	0.23
Fistula	Retinal Vein Occlusion	0.23
Gastroenteritis	Esophagitis	0.23
Gastroenteritis	Peritonitis	0.23
Glaucoma	Abscess	0.23
Graves Disease	Adenoma	0.23
Graves Disease	Pituitary Neoplasms	0.23
Heart Failure	Hemiplegia	0.23
Hemorrhage	Hepatitis, Alcoholic	0.23
Hemorrhage	Hypoglycemia	0.23
Hemorrhage	Sinusitis	0.23
Hepatitis B	Vascular Neoplasms	0.23
Hepatitis B	Herpes Zoster	0.23
Hepatitis B	Nephrotic Syndrome	0.23
Hepatitis C	Infarction	0.23
Hepatitis C	Myocardial Infarction	0.23
Herpes Simplex	Rectal Neoplasms	0.23
Herpes Simplex	Blindness	0.23
Herpes Simplex	Cataract	0.23
Herpes Simplex	Coronary Artery Disease	0.23
Herpes Simplex	Glaucoma	0.23
Herpes Simplex	Infarction	0.23
Herpes Simplex	Myocardial Infarction	0.23
Herpes Zoster	Dehydration	0.23
Herpes Zoster	Glaucoma	0.23
Hyperinsulinism	Liver Cirrhosis	0.23
Hyperinsulinism	Pneumonia	0.23
Hyperinsulinism	Stroke	0.23
Hyperparathyroidism	Multiple Myeloma	0.23
Hyperparathyroidism	Heart Failure	0.23
Hyperparathyroidism	Hyperthyroidism	0.23

Heart Failure	Angina Pectoris	0.61	Hypersplenism	Thrombosis	0.23
Adenoma	Appendicitis	0.60	Hypocalcemia	Cataract	0.23
Adenoma	Thrombosis	0.60	Hypocalcemia	Dehydration	0.23
Fecal Impaction	Diverticulitis	0.60	Hypocalcemia	Thrombosis	0.23
Hepatitis, Alcoholic	Liver Cirrhosis	0.60	Hypoglycemia	Liver Diseases	0.23
Intestinal Pseudo-Obstruction	Intestinal Obstruction	0.60	Hypoparathyroidism	Rickets	0.23
Panniculitis, Peritoneal	Intestinal Obstruction	0.60	Hypotension	Thrombocytopenia	0.23
Esophageal Perforation	Pleural Effusion	0.60	Infarction	Glaucoma	0.23
Fibromuscular Dysplasia	Ischemia	0.60	Infarction	Hyperthyroidism	0.23
Fibromuscular Dysplasia	Stroke	0.60	Infertility	Coronary Artery Disease	0.23
Fibromuscular Dysplasia	Thrombosis	0.60	Intermittent Claudication	Heart Failure	0.23
Heart Failure	Cerebral Infarction	0.60	Intestinal Perforation	Typhoid Fever	0.23
Hemorrhage	Dehydration	0.60	Ischemia	Macular Edema	0.23
Hyperinsulinism	Insulinoma	0.60	Ischemia	Thrombocytopenia	0.23
Hypersplenism	Thrombocytopenia	0.60	Liver Cirrhosis	Rectal Neoplasms	0.23
Hyperthyroidism	Liver Diseases	0.60	Liver Diseases	Sepsis	0.23
Infarction	Abscess	0.60	Macular Edema	Multiple Myeloma	0.23
Infarction	Paraplegia	0.60	Mediastinitis	Meningitis	0.23
Intestinal Perforation	Diarrhea	0.60	Myocardial Infarction	Glaucoma	0.23
Ischemia	Intestinal Obstruction	0.60	Myocardial Infarction	Hyperthyroidism	0.23
Ischemia	Blindness	0.60	Myocardial Ischemia	Q Fever	0.23
Liver Cirrhosis	Pleural Effusion	0.60	Myocardial Ischemia	Pneumonia	0.23
Liver Cirrhosis	Sepsis	0.60	Nephrotic Syndrome	Coronary Artery Disease	0.23
Liver Diseases	Diarrhea	0.60	Osteoarthritis	Adenoma	0.23
Macular Edema	Blindness	0.60	Osteoarthritis	Osteomalacia	0.23
Nephrotic Syndrome	Hyperparathyroidism	0.60	Osteomalacia	Liver Diseases	0.23
Pneumonia	Infarction	0.60	Paraplegia	Adenoma	0.23
Thrombocytosis	Stroke	0.60	Pericardial Effusion	Aneurysm	0.23
Thrombosis	Paraplegia	0.60	Pericardial Effusion	Thrombosis	0.23
Coronary Artery Disease	Myocardial Ischemia	0.58	Peritonitis	Hypotension	0.23
Stroke	Encephalitis	0.57	Pleural Effusion	Multiple Myeloma	0.23
Rectal Neoplasms	Fistula	0.55	Retinal Vein Occlusion	Cataract	0.23
Thrombosis	Hydrocephalus	0.55	Sepsis	Cataract	0.23
Glaucoma	Strabismus	0.54	Sepsis	Congenital Hypothyroidism	0.23
Vitamin D Deficiency	Bone Diseases	0.54	Sepsis	Hyperparathyroidism	0.23
Adenoma	Acrospiroma	0.53	Stroke	Coronary Occlusion	0.23
Adenoma	Adrenocortical Carcinoma	0.53	Stroke	Liver Diseases	0.23
Adenoma	Pneumonia	0.53	Subarachnoid Hemorrhage	Infarction	0.23
Biliary Atresia	Liver Cirrhosis	0.53	Thrombocytopenia	Graves Disease	0.23
Bone Diseases	Osteosclerosis	0.53	Thrombocytopenia	Hydrocephalus	0.23
Brain Neoplasms	Rectal Neoplasms	0.53	Thrombocytosis	Fistula	0.23
Cockayne Syndrome	Cataract	0.53	Thrombocytosis	Pleural Effusion	0.23
Abscess	Discitis	0.53	Thrombosis	Adenocarcinoma, Clear Cell	0.23
Aneurysm	Loeys-Dietz Syndrome	0.53	Thrombosis	Abscess	0.23
Aneurysm	Chest Pain	0.53	Thrombosis	Encephalitis	0.23
Diarrhea	Irritable Bowel Syndrome	0.53	Trachoma	Glaucoma	0.23
Encephalitis	Sepsis	0.53	Ureteral Obstruction	Hydronephrosis	0.23
Fistula	Subarachnoid Hemorrhage	0.53	Ureteral Obstruction	Sepsis	0.23
Graves Disease	Congenital Hypothyroidism	0.53	Vitamin D Deficiency	Hypercalcemia	0.23
Hemorrhage	Angina Pectoris	0.53	Chest Pain	Gastroesophageal Reflux	0.21
Hemorrhage	Hyperthyroidism	0.53	Coronary Occlusion	Infarction	0.21
Hemorrhage	Paraplegia	0.53	Hemorrhage	Diarrhea	0.18
Herpes Simplex	Pneumonia	0.53	Infarction	Meningitis, Bacterial	0.18
Herpes Zoster	Meningitis	0.53	Sepsis	Sinusitis	0.18
Hydronephrosis	Thrombosis	0.53	Appendicitis	Abscess	0.15
Hyperinsulinism	Anovulation	0.53	Adenoma	Encephalitis	0.15
Hypersplenism	Hemorrhage	0.53	Adrenocortical Carcinoma	Hemorrhage	0.15
Hypocalcemia	Sepsis	0.53	Brain Neoplasms	Adenoma	0.15
Hypoglycemia	Ischemia	0.53	Brain Neoplasms	Stroke	0.15
Infarction	Appendicitis	0.53	Discitis	Spondylitis	0.15
Meningitis	Diarrhea	0.53	Fecal Impaction	Fecal Incontinence	0.15
Onchocerciasis	Blindness	0.53	Insulinoma	Hemorrhage	0.15
Pneumonia	Coronary Artery Disease	0.53	Intestinal Obstruction	Diarrhea	0.15
Pneumonia	Thrombocytopenia	0.53	Intestinal Pseudo-Obstruction	Peritonitis	0.15
Sepsis	Thrombocytosis	0.53	Intestinal Pseudo-Obstruction	Thrombosis	0.15
Thrombocytopenia	Hepatitis C	0.53	Meningitis, Bacterial	Cerebral Infarction	0.15
Thrombosis	Cardiac Tamponade	0.53	Meningitis, Bacterial	Sepsis	0.15
Thrombosis	Intermittent Claudication	0.53	Multiple Myeloma	Meningitis	0.15
Vitamin D Deficiency	Heart Failure	0.53	Multiple Myeloma	Sepsis	0.15
Stroke	Subarachnoid Hemorrhage	0.53	Pituitary Neoplasms	Encephalitis	0.15
Sepsis	Blindness	0.51	Protein-Losing Enteropathies	Peritonitis	0.15
Myocardial Infarction	Sepsis	0.50	Protein-Losing Enteropathies	Thrombosis	0.15
Diverticulitis	Appendicitis	0.49	Rectal Neoplasms	Intestinal Perforation	0.15
Intestinal Obstruction	Rectal Neoplasms	0.48	Rectal Neoplasms	Pneumonia	0.15
Sinusitis	Meningitis	0.48	Tuberculosis, Cutaneous	Paraplegia	0.15
Thrombocytosis	Chest Pain	0.48	Typhoid Fever	Abscess	0.15
Brain Neoplasms	Dehydration	0.45	Abscess	Chronic Pain	0.15
Brain Neoplasms	Pneumonia	0.45	Abscess	Hypersplenism	0.15
Mumps	Herpes Zoster	0.45	Abscess	Infertility	0.15
Ascariasis	Intestinal Obstruction	0.45	Abscess	Peptic Ulcer	0.15
Cataract	Strabismus	0.45	Abscess	Pericardial Effusion	0.15
Chest Pain	Esophagitis	0.45	Aneurysm	Gastroenteritis	0.15
Encephalitis	Abscess	0.45	Aneurysm	Liver Cirrhosis	0.15
Endometriosis	Ureteral Obstruction	0.45	Appendicitis	Intestinal Obstruction	0.15
Fistula	Peritonitis	0.45	Cardiac Tamponade	Cerebral Infarction	0.15
Gastroesophageal Reflux	Hemorrhage	0.45	Cardiac Tamponade	Fistula	0.15
Hemorrhage	Blindness	0.45	Cardiac Tamponade	Stroke	0.15
Hemorrhage	Hemiplegia	0.45	Congenital Hypothyroidism	Adenoma	0.15

Hemorrhage	Pericardial Effusion	0.45
Herpes Simplex	Sepsis	0.45
Herpes Zoster	Diarrhea	0.45
Hydrocephalus	Mumps	0.45
Infarction	Nephrotic Syndrome	0.45
Ischemia	Peripheral Arterial Disease	0.45
Liver Diseases	Infertility	0.45
Liver Diseases	Vitamin D Deficiency	0.45
Mediastinitis	Fistula	0.45
Meningitis	Encephalitis	0.45
Myeloproliferative Disorders	Hemorrhage	0.45
Peptic Ulcer	Peritonitis	0.45
Pneumonia	Hypoglycemia	0.45
Sepsis	Adenoma	0.45
Stroke	Rectal Neoplasms	0.45
Thrombosis	Subarachnoid Hemorrhage	0.45
Trachoma	Cataract	0.41
Macular Edema	Cataract	0.41
Thrombosis	Nephrotic Syndrome	0.41
Heart Failure	Hypotension	0.40
Adenoma	Insulinoma	0.38
Adenoma	Hydrocephalus	0.38
Brain Neoplasms	Pituitary Neoplasms	0.38
Carcinoma, Hepatocellular	End Stage Liver Disease	0.38
Carcinoma, Hepatocellular	Ischemia	0.38
Multiple Myeloma	Herpes Zoster	0.38
Rectal Neoplasms	Diverticulitis	0.38
Aneurysm	Adenoma	0.38
Aneurysm	Sepsis	0.38
Blindness	Amblyopia	0.38
Coronary Artery Disease	Peripheral Arterial Disease	0.38
Fistula	Pleural Effusion	0.38
Gastroesophageal Reflux	Peptic Ulcer	0.38
Glaucoma	Hemorrhage	0.38
Graves Disease	Hypocalcemia	0.38
Hemorrhage	Peritonitis	0.38
Herpes Simplex	Herpes Zoster	0.38
Hyperthyroidism	Hypoparathyroidism	0.38
Hypoglycemia	Hypotension	0.38
Infarction	Blindness	0.38
Infarction	Cerebral Infarction	0.38
Infarction	Fistula	0.38
Infarction	Hydrocephalus	0.38
Ischemia	Pneumonia	0.38
Meningitis	Blindness	0.38
Myocardial Infarction	Blindness	0.38
Peritonitis	Myocardial Infarction	0.38
Placenta Accreta	Placenta Previa	0.38
Sepsis	Hydrocephalus	0.38
Stroke	Angina Pectoris	0.38
Subarachnoid Hemorrhage	Meningitis	0.38
Thrombocytopenia	Aneurysm	0.38
Thrombocytosis	Hemorrhage	0.38
Thrombosis	Chest Pain	0.38
Diabetic Retinopathy	Retinal Vein Occlusion	0.38
Abscess	Esophagitis	0.36
Hydrocephalus	Subarachnoid Hemorrhage	0.33
Placenta Accreta	Hemorrhage	0.33
Stroke	Peripheral Arterial Disease	0.33
Adenocarcinoma, Papillary	Adenoma	0.30
Adenoma	ACTH Syndrome, Ectopic	0.30
Adenoma	Bone Diseases	0.30
Adenoma	Focal Nodular Hyperplasia	0.30
Adenoma	Intestinal Obstruction	0.30
Adenoma	Blindness	0.30
Adenoma	Dehydration	0.30
Adenoma	Fistula	0.30
Adenoma	Liver Cirrhosis	0.30
Biliary Atresia	Hemorrhage	0.30
Biliary Atresia	Vitamin K Deficiency	0.30
Bone Diseases	Ischemia	0.30
Bone Diseases	Rickets	0.30
Brain Neoplasms	Abscess	0.30
Brain Neoplasms	Aneurysm	0.30
Brain Neoplasms	Intracranial Aneurysm	0.30
Brain Neoplasms	Strabismus	0.30
Carcinoma, Acinar Cell	Adenoma	0.30
Carcinoma, Hepatocellular	Focal Nodular Hyperplasia	0.30
Carcinoma, Hepatocellular	Hypoglycemia	0.30
Carcinoma, Hepatocellular	Pneumonia	0.30
Chalazion	Adenoma	0.30
Cockayne Syndrome	Hypotension	0.30
Discitis	Ischemia	0.30
Discitis	Meningitis	0.30
Fecal Impaction	Hemorrhage	0.30
Fecal Incontinence	Rectal Neoplasms	0.30
Focal Nodular Hyperplasia	Thrombosis	0.30
Haemophilus Infections	Peritonitis	0.30
Coronary Artery Disease	Chest Pain	0.15
Dehydration	Blindness	0.15
Dehydration	Hypotension	0.15
Dehydration	Meningitis	0.15
Diarrhea	Peritonitis	0.15
Encephalitis	Hydrocephalus	0.15
Endometriosis	Intussusception	0.15
Esophageal Perforation	Esophagitis	0.15
Esophagitis	Rectal Neoplasms	0.15
Fistula	Carcinoma, Hepatocellular	0.15
Fistula	Gastroenteritis	0.15
Fistula	Intestinal Perforation	0.15
Fistula	Liver Diseases	0.15
Fistula	Spinal Cord Ischemia	0.15
Fistula	Stroke	0.15
Gastroesophageal Reflux	Abscess	0.15
Gastroesophageal Reflux	Liver Cirrhosis	0.15
Graves Disease	Hyperparathyroidism	0.15
Heart Failure	Dehydration	0.15
Heart Failure	Diarrhea	0.15
Heart Failure	Hypercalcemia	0.15
Hemiplegia	Herpes Zoster	0.15
Hemorrhage	Amblyopia	0.15
Hemorrhage	Peripheral Arterial Disease	0.15
Hepatitis B	Pleural Effusion	0.15
Hepatitis C	Macular Edema	0.15
Hepatitis C	Peritonitis	0.15
Hydrocephalus	Meningitis, Bacterial	0.15
Hydrocephalus	Herpes Zoster	0.15
Hydrocephalus	Stroke	0.15
Hypercalcemia	Hypocalcemia	0.15
Hyperparathyroidism	Rickets	0.15
Hyperthyroidism	Heart Failure	0.15
Hypoglycemia	Cataract	0.15
Hypoglycemia	Gastroenteritis	0.15
Hypoglycemia	Heart Failure	0.15
Hypoglycemia	Sepsis	0.15
Hypoglycemia	Stroke	0.15
Hypoparathyroidism	Graves Disease	0.15
Hypotension	Cardiac Tamponade	0.15
Hypotension	Meningitis	0.15
Infarction	Carcinoma, Hepatocellular	0.15
Infarction	Cardiac Tamponade	0.15
Infarction	Herpes Zoster	0.15
Intracranial Aneurysm	Ischemia	0.15
Ischemia	Diabetic Retinopathy	0.15
Liver Diseases	Biliary Atresia	0.15
Liver Diseases	Coronary Artery Disease	0.15
Mediastinitis	Esophagitis	0.15
Mediastinitis	Pneumonia	0.15
Mediastinitis	Sepsis	0.15
Meningitis	Thrombosis	0.15
Myocardial Infarction	Abscess	0.15
Myocardial Ischemia	Pleural Effusion	0.15
Nephrotic Syndrome	Graves Disease	0.15
Pericardial Effusion	Meningitis	0.15
Pericardial Effusion	Thrombocytopenia	0.15
Peritonitis	Liver Cirrhosis	0.15
Pleural Effusion	Liver Diseases	0.15
Pneumonia	Esophagitis	0.15
Rickets	Blindness	0.15
Rickets	Heart Failure	0.15
Sepsis	Typhoid Fever	0.15
Sepsis	Osteoarthritis	0.15
Sepsis	Pleural Effusion	0.15
Sinusitis	Meningitis, Bacterial	0.15
Spinal Cord Ischemia	Hypotension	0.15
Stroke	Paraplegia	0.15
Stroke	Sepsis	0.15
Stroke	Sinusitis	0.15
Subarachnoid Hemorrhage	Hypotension	0.15
Thrombocytopenia	Protein-Losing Enteropathies	0.15
Thrombocytopenia	Typhoid Fever	0.15
Thrombosis	Adrenocortical Carcinoma	0.15
Thrombosis	Carcinoma, Hepatocellular	0.15
Stroke	Osteoarthritis	0.13
Abscess	Sinusitis	0.12
Myocardial Infarction	Hypotension	0.12
Stroke	Pneumonia	0.12
Abscess	Peritonitis	0.10
Cerebral Infarction	Meningitis	0.10
Sinusitis	Thrombosis	0.10
Diabetic Retinopathy	Cataract	0.08
Hemorrhage	Heart Failure	0.08
Thrombosis	Myocardial Ischemia	0.08
Adenoma	Abscess	0.08
Adenoma	Heart Failure	0.08
Adenoma	Hypocalcemia	0.08

Intestinal Pseudo-Obstruction	Appendicitis	0.30
Melorheostosis	Osteopoikilosis	0.30
Meningitis, Bacterial	Abscess	0.30
Multiple Myeloma	Intestinal Pseudo-Obstruction	0.30
Multiple Myeloma	Glaucoma	0.30
Multiple Myeloma	Ischemia	0.30
Multiple Myeloma	Liver Cirrhosis	0.30
Multiple Myeloma	Myeloproliferative Disorders	0.30
Multiple Myeloma	Stroke	0.30
Multiple Myeloma	Thrombosis	0.30
Mumps	Hepatitis B	0.30
Neurogenic Bowel	Fecal Incontinence	0.30
Osteosclerosis	Hyperparathyroidism	0.30
Pituitary Neoplasms	ACTH Syndrome, Ectopic	0.30
Pituitary Neoplasms	ACTH-Secreting Pituitary Adenoma	0.30
Pituitary Neoplasms	Carcinoma, Hepatocellular	0.30
Pituitary Neoplasms	Meningitis, Bacterial	0.30
Pituitary Neoplasms	Blindness	0.30
Pyloric Stenosis, Hypertrophic	Intestinal Obstruction	0.30
Q Fever	Blindness	0.30
Rectal Neoplasms	Chronic Pain	0.30
Rectal Neoplasms	Hypersplenism	0.30
Rectal Neoplasms	Intussusception	0.30
Rectal Neoplasms	Ischemia	0.30
Rectal Neoplasms	Peritonitis	0.30
Sigmoid Diseases	Intestinal Obstruction	0.30
Typhoid Fever	Hepatitis B	0.30
Typhoid Fever	Liver Cirrhosis	0.30
alpha 1-Antitrypsin Deficiency	Liver Cirrhosis	0.30
Abscess	Fecal Impaction	0.30
Abscess	Chest Pain	0.30
Abscess	Hydrocephalus	0.30
Abscess	Ischemia	0.30
Aneurysm	Dehydration	0.30
Aneurysm	Diabetic Retinopathy	0.30
Aneurysm	Encephalitis	0.30
Aneurysm	Intermittent Claudication	0.30
Aneurysm	Meningitis	0.30
Aneurysm	Pleural Effusion	0.30
Aneurysm	Strabismus	0.30
Angina Pectoris	Peripheral Arterial Disease	0.30
Anisometropia	Strabismus	0.30
Anovulation	Hemorrhage	0.30
Appendicitis	Hemorrhage	0.30
Ascariasis	Rectal Neoplasms	0.30
Ascariasis	Hemorrhage	0.30
Blindness	Angina Pectoris	0.30
Blindness	Encephalitis	0.30
Blindness	Heart Failure	0.30
Cardiac Tamponade	Dehydration	0.30
Cerebral Amyloid Angiopathy	Infarction	0.30
Cerebral Amyloid Angiopathy	Stroke	0.30
Cerebral Amyloid Angiopathy	Subarachnoid Hemorrhage	0.30
Chest Pain	Rectal Neoplasms	0.30
Chest Pain	Angina Pectoris	0.30
Chest Pain	Diarrhea	0.30
Chronic Pain	Herpes Zoster	0.30
Chronic Pain	Thrombocytopenia	0.30
Congenital Hypothyroidism	Stroke	0.30
Coronary Artery Disease	Cataract	0.30
Coronary Artery Disease	Fistula	0.30
Coronary Artery Disease	Hyperthyroidism	0.30
Dehydration	Gingival Hyperplasia	0.30
Dehydration	Hypoglycemia	0.30
Dehydration	Infarction	0.30
Dehydration	Myocardial Infarction	0.30
Dehydration	Pneumonia	0.30
Diarrhea	Chronic Pain	0.30
Diarrhea	Hemiplegia	0.30
Adenoma	Hypoglycemia	0.08
Adenoma	Sinusitis	0.08
Brain Neoplasms	Endometriosis	0.08
Discitis	Aneurysm	0.08
Pituitary Neoplasms	Heart Failure	0.08
Abscess	Diarrhea	0.08
Abscess	Endometriosis	0.08
Amblyopia	Cataract	0.08
Aneurysm	Hypotension	0.08
Appendicitis	Pneumonia	0.08
Cataract	Anisometropia	0.08
Chest Pain	Heart Failure	0.08
Chronic Pain	Stroke	0.08
Diarrhea	Fecal Incontinence	0.08
Diarrhea	Hepatitis C	0.08
Diarrhea	Thrombosis	0.08
Diarrhea	Trachoma	0.08
Encephalitis	Hypoparathyroidism	0.08
Endometriosis	Thrombosis	0.08
Fistula	Diarrhea	0.08
Heart Failure	Multiple Myeloma	0.08
Heart Failure	Herpes Zoster	0.08
Heart Failure	Peritonitis	0.08
Hemorrhage	Intestinal Perforation	0.08
Hepatitis B	Endometriosis	0.08
Hepatitis B	Herpes Simplex	0.08
Hepatitis C	Vitamin D Deficiency	0.08
Herpes Zoster	Cataract	0.08
Herpes Zoster	Myocardial Infarction	0.08
Hydrocephalus	Fistula	0.08
Hydrocephalus	Paraplegia	0.08
Hyperinsulinism	Liver Diseases	0.08
Hypoglycemia	Blindness	0.08
Hypotension	Intracranial Aneurysm	0.08
Infarction	Thrombocytopenia	0.08
Infertility	Hemorrhage	0.08
Intussusception	Gastroenteritis	0.08
Ischemia	Spinal Cord Ischemia	0.08
Liver Cirrhosis	End Stage Liver Disease	0.08
Liver Diseases	Pneumonia	0.08
Meningitis	Spondylitis	0.08
Meningitis	Peritonitis	0.08
Myocardial Infarction	Thrombocytopenia	0.08
Peritonitis	Aneurysm	0.08
Peritonitis	Infarction	0.08
Peritonitis	Thrombosis	0.08
Pleural Effusion	Abscess	0.08
Pneumonia	Spondylitis	0.08
Pneumonia	Hypotension	0.08
Pneumonia	Pericardial Effusion	0.08
Pneumonia	Thrombosis	0.08
Sepsis	Intestinal Obstruction	0.08
Sepsis	Diabetic Retinopathy	0.08
Sepsis	Vitamin D Deficiency	0.08
Sinusitis	Herpes Simplex	0.08
Spinal Cord Ischemia	Aneurysm	0.08
Stroke	Diabetic Retinopathy	0.08
Stroke	Thrombocytopenia	0.08
Thrombosis	Blindness	0.08
Thrombosis	Liver Diseases	0.08
Trachoma	Diabetic Retinopathy	0.08
Trachoma	Retinal Vein Occlusion	0.08
Vitamin D Deficiency	Thrombosis	0.08
Diarrhea	Pneumonia	0.04
Hemorrhage	Rectal Neoplasms	0.04
Liver Diseases	Bone Diseases	0.03
Typhoid Fever	Aneurysm	0.03
Hemorrhage	Coronary Artery Disease	2.8×10^{-17}

Causal Disease Network of 149 diseases

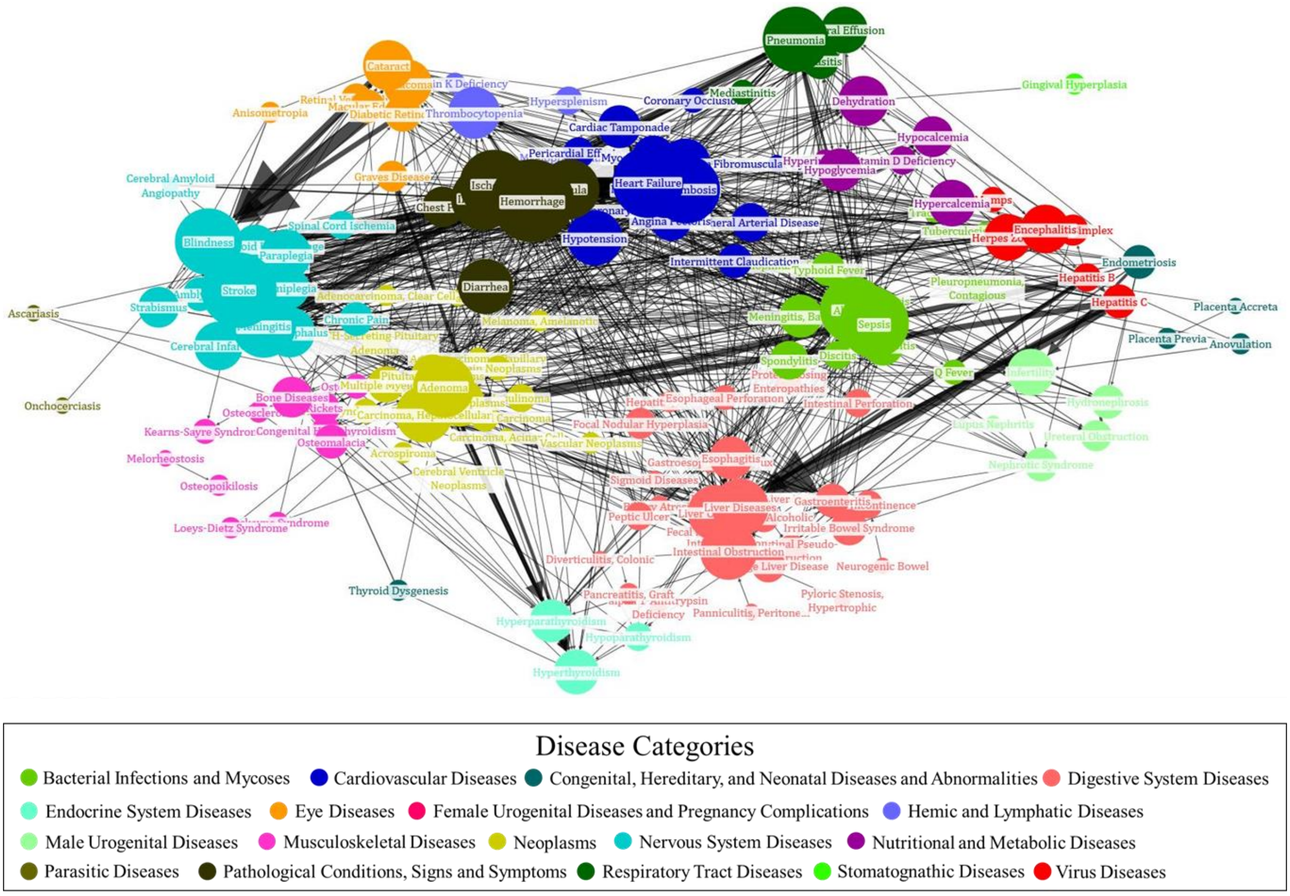

Figure S1. Causal Disease Network of 149 diseases
